# 

**Published:** 1882-12

**Authors:** 


### CONTENTS.

### COMMUNICATIONS.

Dental Prosthesis.................................... 573
Dental Colleges.................................... 578
The Substance that Forms the Skeleton and Teeth—or, Development of the Teeth.................................... 581
Remarks of Dr. C. W. Barrett upon Dental Literature.................... 586
Civilization in its Relation to the Decay of Teeth.................... 589
Treatment of Deciduous Teeth.................................... 601
On Soldering in Dentistry.................................... 607
Mysticism.................................... 611

### CORRESPONDENCE.

From U. Kreit, D.D.S.................................... 615
" E. G. B.................................... 618

### EDITORIAL.

Improvement in Plate Teeth.................................... 619
The Student's Manual of Histology.................................... 621
The Physician's Visiting List for 1883.................................... 622
To the Ohio State Dental Society.................................... 622
Index to Volume XXXVII.

